# Supplementary material for: Perceptions about COVID-19 preventive measures among Ghanaian women
Source: PLoS One. 2023 Apr 12;18(4):e0284362. doi: 10.1371/journal.pone.0284362 (PMC10096443; doi:10.1371/journal.pone.0284362)
Supplement: S1 File — (DOCX) [file pone.0284362.s001.docx]

**Interview guide administered to women**

**Background characteristics**

1. Can you tell me something about yourself?

- Probe: Age, Educational background, Occupation, Marital status, Religion, Number of children, Place of residence (Urban/Rural) etc.

1. Have you been diagnosed with any health condition(s)?

- Probe for chronic non-communicable conditions. E.g., hypertension, diabetes, asthma, heart conditions, etc.

**Knowledge about COVID-19**

1. What is your general perception about COVID-19?

- Probe: How does it spread
- Probe: Prevention of COVID-19

1. Do you perceive COVID-19 as real? Explain your answer
2. Do you think you are susceptible to or at risk of the COVID-19 (re)infection? Why?
3. Do you take any medication to prevent COVID-19 (re)infection? E.g. Taking vitamin C, herbal medication/concoction, etc.

**Adherence to COVID-19 preventive measures**

1. To what extent do people in your community adhere to the COVID-19 protocols?

- Probe: wearing a mask, using alcohol-based sanitizer, hand washing, physical distancing, avoid touching eyes, nose and mouth, avoiding crowded public gatherings or activities, etc.

1. To what extent do you adhere to the COVID-19 protocols?

- Probe: wearing a mask, using alcohol-based sanitizer, hand washing, physical distancing, avoid touching eyes, nose and mouth, avoiding crowded public gatherings or activities, etc.
- If adhering, what is your motivation for adherence?

1. In your opinion, which COVID-19 protocol(s) is/are easiest for people to follow and why?

- Probe: wearing a mask, using alcohol-based sanitizer, hand washing, physical distancing, avoid touching eyes, nose and mouth, avoiding crowded public gatherings or activities, etc.

1. Which COVID-19 protocol(s) is/are easiest for you to follow and why?

- Probe: wearing a mask, using alcohol-based sanitizer, hand washing, physical distancing, avoid touching eyes, nose and mouth, avoiding crowded public gatherings or activities, etc.

1. In your opinion, which COVID-19 protocol(s) is/are difficult for people to follow and why?

- Probe: wearing a mask, using alcohol-based sanitizer, hand washing, physical distancing, avoid touching eyes, nose and mouth, avoiding crowded public gatherings or activities, etc.

1. Which COVID-19 protocol(s) is/are difficult for you to follow and why?

- Probe: wearing a mask, using alcohol-based sanitizer, hand washing, physical distancing, avoid touching eyes, nose and mouth, avoiding crowded public gatherings or activities, etc.
