# Supplementary material for: Perceptions about COVID-19 preventive measures among Ghanaian women
Source: PLoS One. 2023 Apr 12;18(4):e0284362. doi: 10.1371/journal.pone.0284362 (PMC10096443; doi:10.1371/journal.pone.0284362)
Supplement: Attachment — Submitted filename: Response to Reviewers.docx [file pone.0284362.s002.docx]

**Responses to reviewers’ comments**

**Academic editor**

1. The methods section of the study requires review. This is a qualitative study however the design and method used for data analysis is not stated explicitly and not justified. 'Cross-sectional qualitative study' is not an appropriate name; method of sample selection needs to be clearly stated with justification. I recommend the authors use the COREQ check list to guide the reporting of this study.

**Response:**

We have revised the study design and sampling procedure to make study design and sampling procedure understandable. We have also defined convenient sampling procedure in the revised manuscript. See page 5 and 6, lines 108-122.

The method of data analysis was clearly stated in the manuscript. See page 7, lines 153-154.

“The data for the study were analysed thematically using NVivo 10 software following steps recommended by Braun and Clarke [52, 53].”

We have justified why we followed Braun and Clarke recommended steps for analysing qualitative data in the revised manuscript. See page 8, lines 162-164.

“Braun and Clarke recommended steps for analysing qualitative data were followed because it helps researchers to analyse qualitative data systematically and in a rigorous manner.”

We have revised the entire methods section and used the COREQ checklist to revise the reporting of the study.

**Reviewer 1**

1. Reviewer #1: The study is a valuable addition to scientific literature as it used qualitative design. Many studies are available on the subject but only a few utilize interview based approach and present data in that form which is a better reflection of actual barriers that are perceived by the population.

**Response:** We thank the reviewer for the complimentary words.

**Reviewer 2**

1. Page 7, line 153: report the standard deviation and not just the mean age of the respondents under the socio- demographic characteristics.

**Response:** We have reported both the mean age and standard deviation of participants in the revised manuscript. See Table 1 on page 9.

2. Line 155: is there a reference to the categorisation scheme for occupations highlighted in this study or was it arbitrary.

**Response:** The categorisation scheme for occupations was arbitrary based on occupations reported by participants.

3. Page 11, line 177: is the term "dominance" synonymous with frequency of response or agreement.

**Response:** In this study, the term "dominance" is synonymous with frequency of response or agreement.

4. Pages 11 and 12: the reporting of COVID 19 preventive measures as easy to follow versus COVID 19 preventive measures difficult to follow is confusing especially when noting that "wearing a facemask" features as number one in both categories. One would have better understood a spectrum of preventive measures from the easiest to the most difficult. The authors should clarify on this. Another approach recommended could be to take the individual COVID 19 preventive measures and express the relative ease or difficulty in application of each as reported by respondents.

**Response:** We thank the reviewer for the suggestions. We have revised the results on the COVID-19 preventive measures by reporting the preventive measures by their relative difficulty. While reporting the relative difficulty of each preventive measure, we reported on their easiness where applicable. We have also revised the quotes in Table 2 since the quote numbers changed. See pages 12-14 lines 200-238, Table 2.

5. Page 14, Table 3. Quote 2 "As a frontline worker . . . (R11)"
   (Should a frontline ?Health worker, R11, be included in your sample for this study, knowing that her knowledge and job requirement would bias the level of knowledge and lived experience that would be elicited from her compared to other respondents? Can the authors justify her inclusion)

**Response:** The knowledge and job requirement of health workers will bias their level of knowledge and lived experiences. Consequently, three participants were health workers, so we have excluded them from the study. We have changed the sample size in the revised manuscript from 30 to 27. Consequently, we have revised the characteristics of participants, and replaced the quotes of participants who were health workers with quotes of participants who are non-health workers. See page 8, lines 171-177 and Table 1, 2 and 3.

6. Question 4. Yes, the manuscript was reported in Standard English. However the manuscript will benefit from editing such as:
   Abstract
   Page 1, line 39: insert "should" e.g ' . . . measures should continue to be prioritised".

**Response:** We have revised the phrase “measures continue to be prioritised” to “measures should continue to be prioritised”. See page 2, line 40.

7. Introduction
   Page 2, line 60, 61: rephrase ". . . SARS-CoV-2 virus can escape immunity causing infection . . ."

**Response:** We have revised the sentence to “Also, studies have established that SARS-CoV-2, the COVID-19-causing virus, can escape immunity, which can cause infection among people or reinfection among people who have already been vaccinated against COVID-19 or previously been infected with COVID-19 [6, 12-14]”. See 3, lines 60-63.

In addition, we have edited the entire manuscript to address any grammatical error.

8. Methods: Page 5, line 109: The sampling procedure is not clearly explained by the authors. What is "convenient" and "snowballing" sampling procedure in the context of the current study?

**Response:** We have revised the study design and sampling procedure to make sampling procedure understandable. We have also defined convenient sampling procedure in the revised manuscript. See page 5 - 6, lines 108-122.

9. What was the study population and what was so peculiar in recruiting the subjects of the study necessitating the use of snowball sampling?

**Response:** The study population for the study are non-pregnant women aged 18 years or older in the Greater Accra and Ashanti Regions. We have indicated it in the revised manuscript on pages 5-6 lines 112-113.

Also, this study used only convenient sampling rather than convenient and snowball sampling procedures. Therefore, we have deleted snowball sampling from the revised manuscript.
